# An enactive and dynamical systems theory account of dyadic relationships

**DOI:** 10.3389/fpsyg.2014.00452

**Published:** 2014-05-30

**Authors:** Miriam Kyselo, Wolfgang Tschacher

**Affiliations:** ^1^Department of Logic and Philosophy of Science, University of the Basque CountryDonostia-San Sebastián, Spain; ^2^Department of Psychotherapy, University Hospital of Psychiatry and PsychotherapyBern, Switzerland

**Keywords:** enactive self, distinction and participation, dynamical systems theory, well-being in relationships, the self as attractor, relationship dynamics, couples counseling

## Abstract

Many social relationships are a locus of struggle and suffering, either at the individual or interactional level. In this paper we explore why this is the case and suggest a modeling approach for dyadic interactions and the well-being of the participants. To this end we bring together an enactive approach to self with dynamical systems theory. Our basic assumption is that the quality of any social interaction or relationship fundamentally depends on the nature and constitution of the individuals engaged in these interactions. From an enactive perspective the self is conceived as an embodied and socially enacted autonomous system striving to maintain an identity. This striving involves a basic two-fold goal: the ability to exist as an individual in one’s own right, while also being open to and affected by others. In terms of dynamical systems theory one can thus consider the individual self as a self-other organized system represented by a phase space spanned by the dimensions of distinction and participation, where attractors can be defined. Based on two everyday examples of dyadic relationship we propose a simple model of relationship dynamics, in which struggle or well-being in the dyad is analyzed in terms of movements of dyadic states that are in tension or in harmony with individually developed attractors. Our model predicts that relationships can be sustained when the dyad develops a new joint attractor toward which dyadic states tend to move, and well-being when this attractor is in balance with the individuals’ attractors. We outline how this can inspire research on psychotherapy. The psychotherapy process itself provides a setting that supports clients to become aware how they fare with regards to the two-fold norm of distinction and participation and develop, through active engagement between client (or couple) and therapist, strategies to co-negotiate their self-organization.

## INTRODUCTION

Many social relationships are a locus of struggle and suffering, either at the individual or interactional level. Dyadic exchange and the question of well-being in relationships constitute the core of the psychotherapeutic process, as well as the content of most narratives processed in everyday life. Our goal is to better understand why some couples manage to sustain their interactions whereas others terminate their relationships. We also wish to generate ideas for improving the quality of dyadic interactions and the psychological well-being of the participants. To this end we conjoin a dynamical systems theory perspective with an enactive approach to self and explore the dynamics underlying struggle in couples’ relationships.

Dynamical systems theory (DST) is a branch of mathematics, and as such neither part of the natural sciences nor of the humanities (Salvatore and Tschacher, 2012). Its concepts, heuristics and methods can be used to interrelate theories and findings of the various disciplines and to facilitate the dialogue between them. DST describes the complex behavior of systems over time. It allows us to interrelate experiential findings associated with relationship struggle and to derive implications for improving dyadic interaction and enabling relationships.

However, before assessing problems at the level of the interaction we should clarify our understanding of the individuals involved in it. We need to reconsider their basic nature as individuals and what drives their behavior. We suggest characterizing the individuals in the dyad from an enactive perspective, according to which every individual self is genuinely social and purposeful. The enactive self is social because it exists through engagements with others, and it is purposeful because it thereby strives to survive as a social existence. The self follows a primordial two-fold existential norm: being distinct from, as well as connected to, others. We propose that such a basic normative structure of self exists in all individuals. It guides their behavior and how they evaluate and negotiate their relationships.

Our strategy is as follows. We begin with a brief summary of the enactive self in Section “Distinction and Participation: An Enactive Approach to Self.” Based on this, as an intermediate step, we conceptualize in Section “Socially Enacted Autonomy from a Dynamical Systems Theory Perspective” the enactive self in terms of dynamical systems theory as a non-linear dynamical system. In Section “Dyadic Relationship as Negotiation of Individual and Dyadic Attractor Regions” we introduce two everyday examples of couple relationships using our concepts to describe the dynamics underlying the struggle in these interactions, and to arrive at two simple models of relationship maintenance. In Section “Discussion” we compare the two examples and derive two styles of individual relationship engagement, the passive-closed and active-open style, hypothesizing that the latter is more apt to sustain a relationship and to improve well-being in a dyadic relationship. In the last part we outline how the findings in this paper may inspire research in psychotherapy.

## DISTINCTION AND PARTICIPATION: AN ENACTIVE APPROACH TO SELF

In this section we provide a short summary of the enactive approach to self as a social autonomous system (Kyselo, submitted), a recent development in enactivism. Enactivism is a non-reductionist and integrative epistemological framework for cognitive science that adopts a process-based and biologically grounded perspective on cognition (Varela et al., 1993; Jonas, 1966; Varela, 1997; Weber and Varela, 2002; Thompson, 2007). It is rooted in the theory of autopoiesis and the idea that living beings can be minimally characterized as self-producing and self-organizing networks of biological processes that create a systemic identity (Maturana and Varela, 1980, 1987). Enactivism assumes that biological and mental phenomena are continuous and that therefore the identity of cognitive beings can be conceived as based on similar principles and concepts (Clark, 2001; Thompson and Varela, 2001; Di Paolo et al., 2010). It is thus inspired by the autopoietic idea of self-generated identity, but elaborates on this concept by suggesting the more general notion of *autonomy* to capture not only biological but also cognitive individuation (Di Paolo, 2005; Thompson, 2007; Barandiaran et al., 2009; Di Paolo et al., 2010). In the enactive view on autonomy there is no clear-cut separation between individual system and environment. Cognitive individuals emerge from active engagement with the environment through which they self-produce an identity. They thereby follow an intrinsic purpose, namely to survive and to maintain their self-generated identity (Weber and Varela, 2002). This implies a basic *tension* in the individual: a need to emancipate oneself from the environment as an individual, while at the same time having a structural dependence on it for material resources (Jonas, 1966).

Through being self-organized in this way, individuals always have their own basic perspective on the world, i.e., they evaluate their interactions with the world according to what these interactions mean with regards to the goal of generating and maintaining an identity. The enactivists call this sense-making, the value-driven active engagement with the environment that in turn creates meaning for the system itself (Weber and Varela, 2002; Thompson and Stapleton, 2009).

The enactive view on cognitive individuation has been recently elaborated to inspire a new look at the human self (Kyselo, submitted). According to this, the self is essentially a phenomenon of life and a question about the nature of human cognitive individuation. Usually the processes of cognitive individuation have been characterized in terms of embodiment (Kyselo and Di Paolo, 2013; Di Paolo and Thompson, forthcoming), but according to the enactive perspective on the human self the body is not the sole source of individuation. The world of humans is a world of others, so our social relations are what matter most to us. Much in line with theories of self that emphasize the social, processual or dialogical nature of self (Mead, 1934; Buber, 1947/2002; Vygotsky, 1986; Hermans et al., 1992; Tschacher and Rössler, 1996; Mahler et al., 2000) the enactive approach thus assumes that the social must play a vital role in any description of human cognitive individuation.

The enactive self is operationally defined as a *socially enacted* autonomous system, whose systemic network identity emerges as a result of an ongoing engagement in social interaction processes that can be qualified as moving in two opposed directions, *distinction* and *participation* (Kyselo, submitted). On this account, the self as identity is continuously co-generated through interacting and being related to others and at the time organizes interactions and relations. The individual self is therefore never fully separable from the social environment. It is determined precisely in terms of the types of social interactions and relations of which it is also a part. Yet in order to exist as an identifiable unity, the self also involves an ongoing process of emancipation from others. This basic tension between dependence and emancipation is primordial to the nature of human individuation, and for this reason, it is considered a fundamental drive for human behavior. Whereas living systems strive to survive by avoiding interactions with the environment that threaten their biological survival, the purpose of the human self is additionally to ensure its identity and survive as *social existence*^1^. In line with the enactive perspective on autonomy, every individual self thus has its own subjective perspective on the world, a perspective from which social interactions and relations are evaluated according to whether and how they serve the survival, i.e., maintenance, of the self. This maintenance follows a two-fold basic norm that mirrors the tension of the social individual to emancipate itself from the social environment while at the same time structurally relying on it: being able to exist as individual in one’s own right (*distinction*) while at the same time remaining connected with others (*participation*). Distinction means that the person experiences herself as both emancipated and yet not fully independent of the social world. Participation means that she feels both connected and open to, but also not fully immersed in, the social world. Both dimensions can overlap. Distinction does not imply that the person does not interact or has to be alone (think of the familiar experience of feeling alone despite being surrounded by others). Participation, on the other hand, does not imply that the person must interact all the time. A person can feel very open or related to another person even when not actually engaged with her. Both distinction and participation are (experienced) types of social interactions and relations, yet they may say little about the amount or actuality of engagement. In every individual the amount and distribution of distinction and participation can come in different degrees: some individuals have a generally strong sense of being an individual in their own right, but feel not so open to others, while others feel equally open to others.

Throughout an individual’s life there are phases when one may feel or strive for more or less distinction or participation; as a child, for instance, there might be a stronger openness to being affected by others and a lower experience of being separated, whereas during adolescence feelings of being or wanting to be separated are more dominant. Distinction or participation furthermore depend on a given cultural context, and on whether a greater value of one or the other is developed because it is socially more accepted (Markus and Kitayama, 2010). Furthermore, even though at times one of the dimensions may become extremely dominant and the other appears out of reach, the other dimension can or will, at some point or implicitly, drive the individual’s behavior. Thus, for example, feeling very distinct at some point does not mean that there is no striving for connection and openness anymore.

An excessive degree of distinction would mean that the individual has no sense of openness or connection to others, while excessive participation would mean that the individual is completely immersed in the interaction. Humans thus strive to avoid the double risk of emancipation at the cost of being isolated and of connection at the cost of dissolution of the individual self. We can find examples that approximate such extreme cases in disorders of the self such as schizophrenia (Parnas and Sass, 2010) and symptoms like social or self-isolation (no participation) or loss of agency (no distinction). But even though these cases are exceptional, the suffering that accompanies these extreme states could actually be indicative of a persisting striving to balance both dimensions.

Importantly, the maintenance of the self according to the two-fold normative structure of distinction and participation requires constant negotiation with others, that is, engaging with, and disengaging from them. The self is thus co-generated with others and since interactions with others can go wrong and fail to contribute to identity maintenance in the desired way, the self is genuinely vulnerable.

In the next section we conceptualize this view on self in terms of dynamical systems theory and then derive a model of interaction dynamics between two selves.

## SOCIALLY ENACTED AUTONOMY FROM A DYNAMICAL SYSTEMS THEORY PERSPECTIVE

Dynamical systems theory (DST) allows describing a system at two levels: by variables that denote state changes of systems over time, and by parameters that constrain these changes (gradients). DST has been used in cognitive science to replace the input/output model of cognition, and propose a context- and time-sensitive account of cognition (Thelen and Smith, 1994; Van Gelder, 1998; Tschacher and Dauwalder, 1999) and neural dynamics (Haken and Tschacher, 2010). More recently, researchers in enactive cognitive science have appealed to DST to describe mind, social interaction, and sensorimotor skill-use (Thompson, 2007; De Jaegher and Di Paolo, 2007; Froese and Di Paolo, 2010; Buhrmann et al., 2013). In this section we use DST to conceptualize the self as socially enacted autonomy.

We begin with a brief reminder of some of the main concepts of DST. A core notion especially relevant for our purposes is the concept of *attractor,* which can be formally defined in terms of the *phase space* of a system. The phase space is a geometrical space with one or more dimensions, depending on the number of variables needed to fully describe the system (Abraham and Shaw, 1992). This can be exemplified by Euclidean space: Euclidean phase space has three spatial dimensions, i.e., the coordinate axes *x*, *y*, and *z*. Any state of an object situated in Euclidean phase space can simply be described by the three spatial coordinates. DST captures not only a particular state of the system, but also how the system changes in time. 

Imagine a golf ball being hit, flying through space, landing on grass, rolling and ending up in a well or the hole of a golf course. The golf ball represents a simple dynamical system. While flying through the air, rolling on the grass, etc., the ball follows a specific *trajectory* (its flight or path) through Euclidean space (the three dimensions of space). Until it ends up at the bottom of a hole, the ball trajects the three dimensions of space (*x, y*, and *z*), changing its states over time. The state of the ball in the hole, after it has come to a rest, can be represented as a particular single point in phase space, to which the three dimensions of the system’s trajectory through space have converged. Assuming that the ball will always end up in the hole after many different trajectories, we would arrive at a simple illustration of a dynamics in which the system’s three dimensions are always compressed toward zero dimensions, which is indicative of so-called attractor dynamics. The unchanging, stable state, such as in the bottom of the hole in golf, is referred to as a *point attractor.*

As the example of the golf ball illustrates, DST concepts allow visualizing complex temporal behaviors of particular systems in terms of geometrical representations. We use the notions of *phase space*, *trajectory* and *attractor* to conceptualize the states and changes of a human self.

From a DST perspective the human self can be seen as a non-linear dynamical system that displays a particular behavior represented as movement through the “landscape” of *phase space* (Nowak et al., 2002; Tschacher and Munt, 2013). The phase space of the enactive self refers to the states of the self as created in its relations with the social environment. It consists of representations of social interactions and relations, covering idealized engagements with and disengagements from others throughout a life-time. The self’s phase space is therefore a space of the two fundamental variables of *distinction* and *participation* as introduced in the previous section.

In order to define the phase space of an enactive self at the most *general* level we abstract over all possible variations of distinction and participation (individually preferred ranges, different cultures, at different times of life) and use distinction and participation as variables *D* and *P*. This is in line with abstract conceptualizations of psychological phenomena, such as Kurt Lewin’s topological psychology (Lewin, 1936), in which personality and social relations are modeled in terms of regions and barriers in ‘life space’ (Tschacher and Dauwalder, 1999). Our model of phase space may also be associated with theoretical psychology (Leary, 1957), according to which personality involves an interpersonal space that is similarly constituted by two dimensions, agency and communion (Horowitz et al., 2006). In terms of psychological development this resonates with the work of Mahler, who described the infant’s self development as a process of individuation and separation through which the infant’s self emerges subsequent to a post-natal period of symbiotic relation with the mother (Mahler et al., 2000).

**FIGURE 1:**
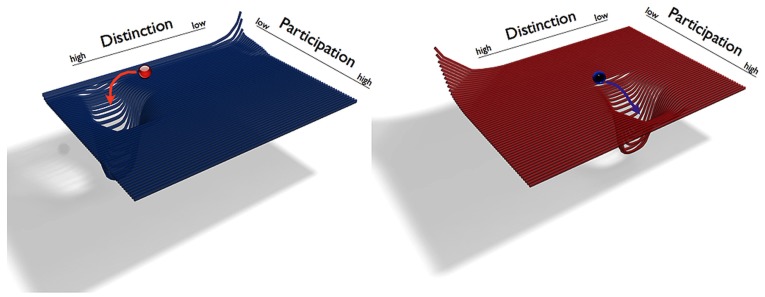
**An illustration of two generic individual phase spaces of the enactive self.** The individual self states are symbolized as red and blue balls. Their respective phase spaces are spanned by the variables of Distinction *D* and Participation *P*. The attractors are wells in *D/P* landscape, into which the self systems move. The red and blue arrows indicate the trajectories of these movements. The graphic on the left side illustrates a self (red ball) with an attractor with a greater value of *D* and a lower value of *P*. The graphic on the right side illustrates a self (blue ball) with an attractor with a relatively high value of P and a lower value of *D*.

The variables *D* and *P* span the self’s phase space, which can thus be illustrated as a plane (**Figure 1**). We denote the states of the self by their locations in this plane (*D/P*). The higher the value of *D*, the higher the system’s distinction, and vice versa, the higher the value of *P*, the higher is the system’s participation. Since the enactive self is always relational, neither *D* nor *P* can ever have a value of zero. In addition to its value of *D* and *P*, each point (*D/P*) of the plane has a positive or negative “elevation,” so that the corresponding slope represents repulsion from or attraction toward this point. The self’s behavior is represented by trajectories, i.e., sequences of states in the phase space. Over time a self develops particular tendencies to balance *D* and *P* (becoming more or less distinct from others and more or less open to them). In terms of DST we can say that the trajectories tend toward particular regions of the phase space. When these tendencies become manifest, these regions can eventually exhibit attractors we define in terms of a particular value *D/P*. The attractor regions symbolize the location of the individual’s developed and preferred zones of functioning (balancing *D* and *P*). The phase space of each self has regions with negative elevations (“wells” or “troughs” in the landscape of phase space) that indicate attractors toward which the system will move. Some regions are positively elevated and thus repulsive, which indicates so-called repellors (the opposite of attractors). Attractors and repellors emerge due to habitual tendencies and represent goals of the self throughout a particular time span. Attractors of distinction and participation must not mean that the individual evaluates them as positive. Such tendencies and goals may emerge due to the individual’s increased well-being in that region, but they may also emerge due to system-external reasons (not controlled by the individual) or habitual tendencies with a negative connotation.

Attractors may exist in different regions with different values *D/P.* They represent that an individual self has developed or exhibits a certain degree of distinction in combination with a certain degree of participation. Consider a person who developed a strong preference for high distinction and low participation for particular life times. For example, a novelist who at some point during the writing process escapes her social life, locks herself up in a remote and quiet place in the mountains to finish her new book. The novelist has experienced this kind of solitude as useful, and so whenever she writes a book, she retreats to the cabin. In terms of DST, during the book writing phases the novelist’s phase space shows a particular attractor *D/P* with a higher value of *D* and a lower value of *P* than usually. We may imagine the system starting at some point in *D/P* landscape. The inclination of the landscape at this point will then determine the direction of the trajectory, which is generally away from repellors and toward the deepest points of attractors. The system will change its distinction and participation values until it has reached the point attractor *D/P* (solitude and a minimum of engagement with others). The system will remain in the attractor region unless the phase space changes or until perturbations external to the system exert an influence.

To describe how systems’ tendencies change through perturbation, we can refer to another concept in DST, the gradients. Gradients are often referred to as control parameters, but in the context of dynamics of the self’s organization this term, borrowed from physics, is misleading. Gradients here are the environmental fluxes and affordances that drive, but do not control, a system’s self-organization. At certain critical values of a gradient, a system may enter a novel, emergent state of its dynamics, and hence its phase space landscape may become completely modified. Because of these changes new attractors may arise, so that the phase space of the enactive self should be conceived of as a flexible landscape. In case of the enactive self, gradients can refer to the social environment. Imagine for example that during the writer’s exile a friend in need reaches out asking for support in a difficult matter. This perturbates and might also change the novelist’s current states in *D/P* landscape. In tension to her initially preferred region of low *P* she reacts to the friend’s perturbation by turning toward a region with higher *P* values, thus adapting the current range of preference in *D/P*. Another example is attending some party or other obligatory social gathering, when one would actually prefer spending a quiet evening at home. Because the social event requires higher values of *P* it can signify a strong perturbation to the current disposition of *D/lower P*. The prospects of attending the party can therefore cause tension and actual struggle while being there.

Strong emotions and other motivational parameters may also be gradients that affect the self. For example, when the book is finished, the novelist’s attractor may shift back to a different region, with a higher value of participation. Here the gradient is motivational (the author realizes that the book is finished). Persistent sadness may for instance change a person’s *D/P* landscape and entail avoiding connection with others, shifting her states toward lower *P* values. Gradients can thus perturbate and change the values of the self’s attractor.

**FIGURE 2:**
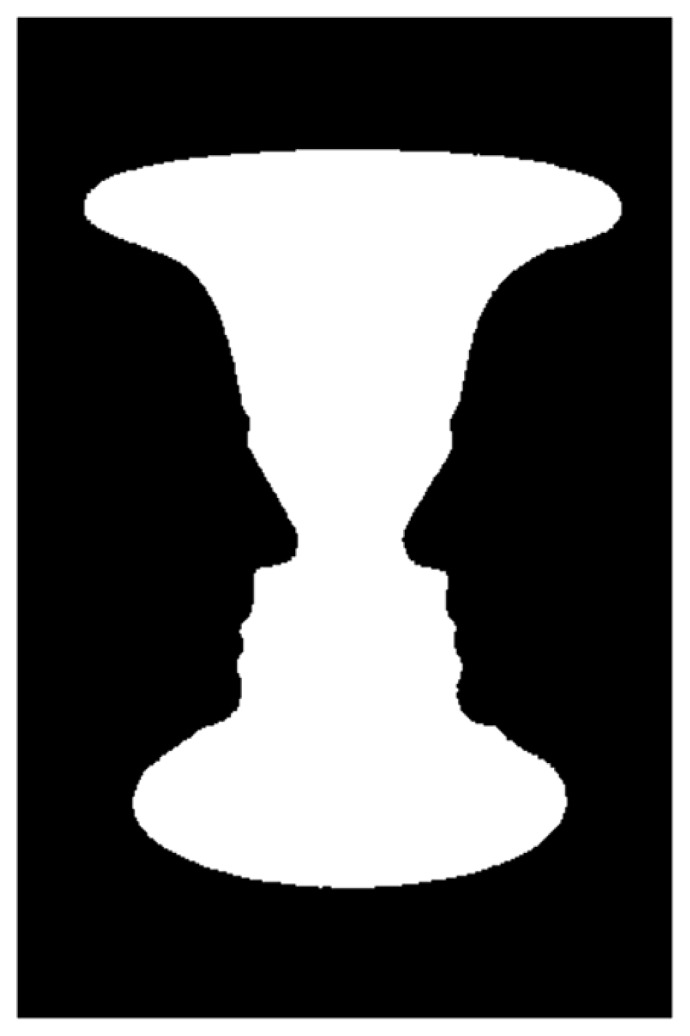
**A bistable stimulus according to Rubin (1921)**.

A formerly active attractor may “close,” leaving behind a quasi-attractor in the same location (Haken, 2006). An example of this is the perception of bistable stimuli, such as Rubin’s vase-face figure (**Figure 2**). When the first perception is that of a white vase, i.e., the perceiver rests in the attractor “vase,” this will eventually give way to the new perception “black faces.” In terms of DST, the system has altered its display of attractors and the landscape of phase space has changed (Haken, 1992). By staying in one attractor (“vase”), the attention gradient that created this attractor becomes depleted (Tschacher and Haken, 2007), so that the system will explore other regions of phase space to eventually settle in a different attractor (“faces”).

To sum up, we conceptualized the enactive self in terms of DST as an attractor landscape in a social phase space that is organized by the two variables of distinction and participation. Individual changes of distinction and participation are expressed in terms of trajectories through this landscape, and strong behavioral tendencies in terms of attractors *D/P*. The stability of such patterns of the self are constantly maintained and calibrated by external and system-inherent gradients, such that changes of gradients will generally change the self’s whole landscape. In the following section we explore two examples of dyadic interactions on the basis of these considerations.

## DYADIC RELATIONSHIP AS NEGOTIATION OF INDIVIDUAL AND DYADIC ATTRACTOR REGIONS

In this section we use the conception of the individual self in terms of a *D/P* attractor dynamics for understanding dyadic relationships. We will introduce two everyday examples of relationship struggle, one in which interaction leads to a breakup the other in which interactions are sustained. We conceptualize the two couples in terms of DST as a dyadic relation between two individual phase spaces. That dyadic relationship can be described as a new kind of dynamical autonomous system (Luhmann, 1992; De Jaegher and Di Paolo, 2007). We conceive of it as a new dynamical system with a phase space that corresponds to sustained interactions between the individuals in the relationship, a joint phase space.

For reasons of simplicity, we assume that the formation of the couple’s joint phase space is a summation of the phase spaces of the individuals: we thus add the elevation values of the individual phase spaces in each point of *D* and *P*. This means that when both participants previously had an attractor in the same region of their individual phase spaces, their dyadic joint phase space will have an even deeper attractor in this region.

We then assume that at each point in time the states of the interaction dynamic, represented through particular locations in the dyad’s phase space, affect the partners, in that they act as perturbations on their individual phase spaces *D/P*. Such perturbations occur at all times during the relationship. It will be a task for the future to elaborate more concrete structures, but we offer a first idea of how a joint state could affect an individual. Firstly, interactions can perturbate one or both dimensions of the individual’s developed or preferred range *D/P*, distinction and/or participation (they can act as gradients). Secondly, not every perturbation must lead to change in a current state or developed attractor *D/P.* Thirdly, it will depend on the frequency and the quality of particular interactions or patterns of interactions whether and how each state or attractor is affected. We can assume that for each dimension *D* or *P* there will be interaction qualities that currently matter more or less. For example, interactions that are too frequent and aggressive, or not frequent and gentle enough, may perturbate stronger on the dimension of *P* (*openness*) in some individuals, while interactions bringing forth a pattern of belittlement and shame on the one hand, or praise and recognition on the other, may be more relevant to the dimension of *D* (*distinction)*. Whether and how much of the quality of any of such interactions perturbates *D* or *P* depends on the individuals. In the following conceptualization of two case examples in terms of DST we chose to refrain from more precise description and restrict the analysis to a fairly general level of interrelating individual and joint action. It will provide a very basic answer to our question: why do couples struggle and what constitutes well-being in a relationship? Each example is approached based on two basic questions: firstly, how the individuals’ particular negotiation tendency, i.e., their respective range of distinction and participation initially match, and secondly, whether and to what extent the actual interaction allows the participants to maintain or to negotiate their individual goals of balancing *D* and *P.*

**Example 1**

She, an artist, has been exploring her inner experiential world continuously in recent years investing considerable time and effort in various practices of mindfulness such as yoga and meditation. Although she is, as a performing artist, used to present herself on stage, she is careful about the exhibition of her private self outside of the roles on stage. She is generally rather inhibited to engage in an intimate relationship. He, a scientist, is used to communicate his personal projects in public and has a strong communion motif privately, being eager to engage in an intimate relationship. After the two met at a workshop and with him taking the initiative, the two soon enter an intense romantic relationship. The initial months of the relationship are full of frequent meetings in a highly participatory mode. Soon, after a few months however, she begins to feel pressured and cornered by him. She experiences fears and crises, mainly as attempts to emancipate herself from the risk of becoming too dependent on him. “Isn’t it true that people should learn to love themselves first?” she asks. To accommodate her struggle the couple decides to try a more detached and individualized style of relationship allowing her to spend more time by herself. After a while however, he begins to feel unhappy about the lack of frequency and intensity of their meetings. In his view they do not see each other enough. The next months of the relationship show a continuous oscillation between attempts to accommodate her need for more time by herself and his need for more time being together. While he experiences her effort for emancipation as too high, she experiences his effort for being together as too much. She insists that over-attachment to the other is not love. He complains that she is pushing him away. They have repeated arguments about the meaning and goals of a relationship, their attempts at improving the relationship do not reach consensus resulting in continuous emotional dissonance for both of them. The couple eventually splits up after about 1 year of being together.

Let us begin with the first question, the individuals’ general tendencies of interrelating *D* and *P* with regards to the prospective romantic relationship *before* they enter the relationship. Based on the above case we derive that she has a stronger tendency toward distinction and toward a sense of self as being a separated individual, whereas his profile shows tendencies in the opposite direction, toward a more participatory mode of identity construction. We can state that the individuals’ attractors dwell in different regions of phase space: as an individual, and with regards to romantic relationship, her attractor resides in a region with a greater value of *D* and a lower value of *P*. His attractor is in a region with low distinction and high participation, featuring a lower value of *D* and a greater value of *P*. Whenever these two individuals start from mid-range values of distinction and participation, the joint trajectory heads in opposition to her or his previous trajectory. This couple corresponds to the phase spaces of the example that we have given in the previous section “Socially Enacted Autonomy from a Dynamical Systems Theory Perspective” (**Figure 1**).

**FIGURE 3:**
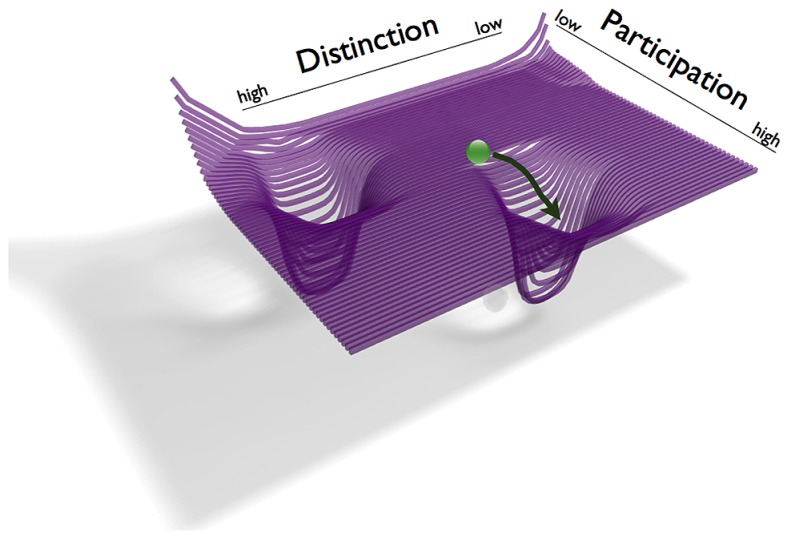
**Illustration of the joint phase space of the couple in *example 1.*** The dyadic phase space is a summation of the individual phase spaces (his low *D* and high *P* and her high *D* and low *P,* see **Figure 1**). The graphic illustrates the couple’s states during the initial months of the relationship (*t1*). The green ball symbolizes that they move in a region with higher values of participation.

Let us now consider what happens when the two individuals in the couple of *example 1* enter a relationship. To this end we thus create a joint phase space adding the individuals’ preferred attractors of *D/P*. The new dyadic phase space thus exhibits two attractors that correspond to the former individual attractors (**Figure 3**). Dividing the time of their relationship into temporal windows, we look at three states this coupled system goes through: *t1*, initial months; *t2*, adjustment phase I; *t3*, adjustment phase II.

At *t1,* corresponding to the closeness and intensity experienced in the initial phase of their relationship, we see that the dyad’s states tend to reside in a range close to high values of participation and lower of distinction (**Figure 3**). This is in accordance with his previous individual attractor that showed a greater value of participation. It is in dissonance with her previous attractor that had a higher value of distinction.

At *t2* the dyad’s state resides in a new region within *D/P* plane migrating to an attractor with higher distinction and lower participation levels. At the individual level this means that the dyad’s trajectory thus moves closer to her individual range of preference and farther away from his. However, the system does not remain in this region but moves back again to the previous region of higher distinction and lower participation levels, in accordance with her and in tension with his individual preference.

**FIGURE 4:**
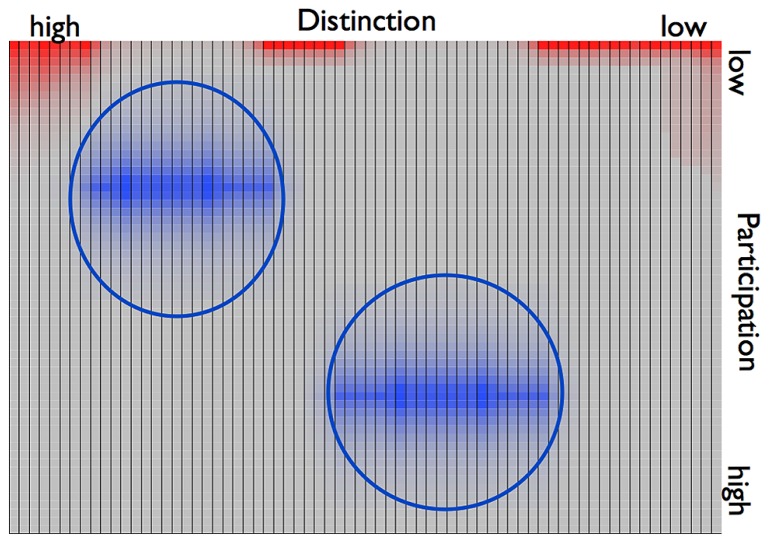
**2D illustration of the joint phase space of the couple described by *example 1*.** The attractors are depicted in blue, the repellors in red. Attractors are circled showing that there was no overlap of the basins of the two attractors.****

Subsequently, during the adjustment phase II, the dyad’s states keep oscillating between the two opposite attractors. Except for *t1* the trajectories of the dyad never persistently overlap with the individually preferred ranges (**Figure 4**). One might describe this behavior in terms of a bistable quasi-attractor dynamics, as in the example of **Figure 2**. The dyad depletes a current attractor and subsequently revives a former quasi-attractor in the sense of Haken (2006), to then again deplete it and revive the previous one. The couple’s transients between the two attractors eventually result in a collapse of the system at *t3*.

**Example 2**

She, an artist, has been exploring her inner experiential world continuously in recent years investing considerable time and effort in various practices of mindfulness such as yoga and meditation. Although she is, as a performing artist, used to present herself on stage, she is more careful about the exhibition of her private self outside of the roles on stage and more reluctant to engage in an intimate relationship. He, a scientist, is used to communicate his personal projects in public and also has a strong communion motif privately being eager to engage in an intimate relationship. However, he also likes spending a lot of time by himself. Since the beginning of their relationship the couple experiences short-lasting but intensive crises. In these crises she feels pressure and fears of being overwhelmed and losing control. She would like to be by herself but at the same time she does not want to leave the interaction, afraid to lose the connection or to hurt him. He usually is shocked at the expression of her discontent and feels overwhelmed or afraid of failing to please her. At the same time he also experiences a strong pull to stay in the situation with her, either because he is afraid to hurt her or to lose her. Both are convinced that an intimate relationship requires efforts on both sides and so they try different strategies to deal with their crises. Occasionally the couple decides to briefly interrupt the interaction trying to become aware of individual feelings without worrying what the other does. At other times, overcoming feelings of panic and losing control, they are open and trusting toward the other and remain in the interaction. Both experience these phases as difficult and feel strong emotional dissonance. But they also learn that momentary disconnection does not necessarily threaten their relationship and that what initially seemed frustrating can actually lead to a better mutual understanding. The couple experiences this as nourishing and as deepening their connection.

**FIGURE 5:**
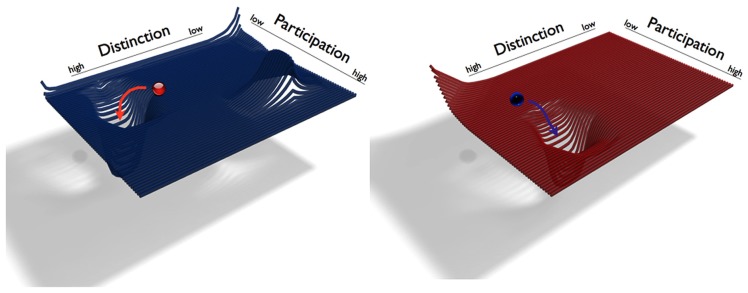
**Illustration of the phase spaces of the individuals in *example 2* (left: “She”; right: “He”).** The phase spaces are spanned by the dimensions of Distinction *D* and Participation *P*. Individual system states are symbolized as locations of red and blue balls. The attractors are wells in the *D*/*P* landscape, into which the self systems (balls) tend to move. Arrows indicate trajectories from two arbitrary starting points.

Let us begin with the first question, the individuals’ general tendencies of interrelating *D* and *P* with regards to the prospective romantic relationship *before* they enter the relationship. Based on the above case we derive that she has a strong tendency toward distinction and toward a sense of self as being a separated individual whereas his profile shows tendencies in the opposite direction, toward a more participatory mode of identity construction. However he also shows relatively high tendencies toward distinction. The individuals thus have different preferences in negotiation of distinction and participation, i.e., the attractors of the individuals are in different, but not opposite regions of phase space: she has a *high D*/*low P* attractor, and a repellor at *low D/high P*. The repellor represents her inhibition for highly participatory states when the range of distinction is low. His attractor is also at greater values of *D* together with moderate to *high P* (**Figure 5**, please note that her attractor is identical to the attractor of the “she” protagonist of *example 1*, cf. **Figure 1** left), whereas his attractor slightly differs in the two narratives.

**FIGURE 6:**
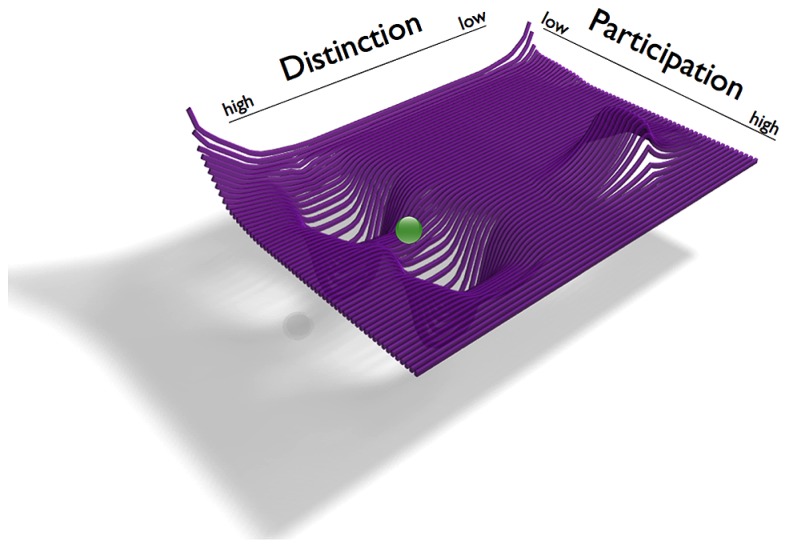
**Illustration of the dyadic phase space of *example 2*.** The dyadic phase space corresponds to the individual phase spaces from **Figure 5**. The couple’s state (green ball) is located in the saddle, the region connecting the individual attractor regions.

**FIGURE 7:**
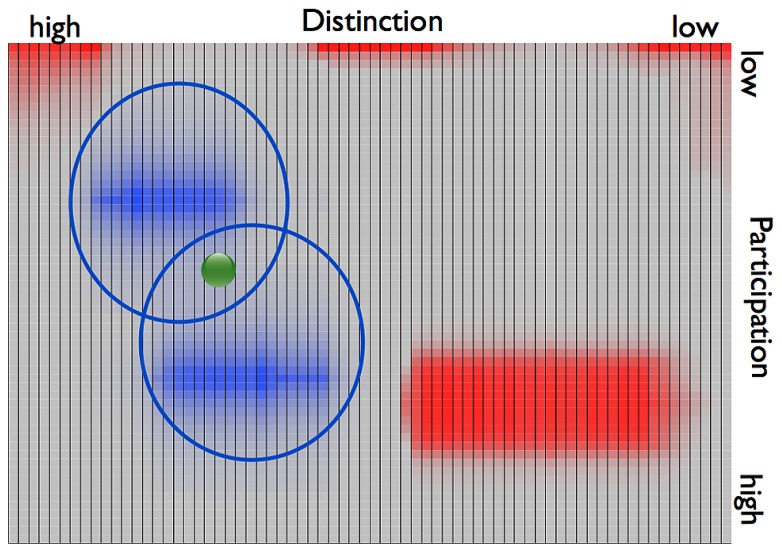
**2D plane view on the dyadic phase space of *example 2.*** The two attractor regions are depicted in blue, the repellors in red. The attractors are circled. Their inters

Let us now describe the situation once the individuals of *example 2* enter a relationship and the individual phase spaces are merged into one joint phase space (**Figure 6**). Corresponding to the couple’s several instances of crises, the dyad’s states in *example 2* oscillate between the two attractor regions. The dyad’s behavior thus shows similarity to that of *example 1.* However, the transients between the “deepest” points of the attractors here are considerably shorter than in the dyad of *example 1*. Even though the oscillations occur between different levels of participation, the individuals show an overlap in their previous attractors with a high value of distinction. The couple in this example thus has a region in which the individuals share individual preferences. In terms of DST this is to say that the basins of the two individual attractor regions create an intersection, i.e., a region of overlap (**Figure 7**). Such connections between point attractors are called “saddles” (**Figure 6**). If the couple continues to sustain interactions leading to an overlap of their attractors, a saddle could “deepen” and turn into a new, jointly created attractor indicating the couple’s sustained interaction tendencies.

Conceptualizing the two relationship examples in terms of dyadic movements away and toward greater distinction or participation that are either in accordance with or deviating from the individually developed attractor, we offer a simple model of co-negotiation of self maintenance in dyadic interaction. In the following section we compare the two couples and discuss what the observed state changes could mean for relationship sustainment and individual well-being.

## DISCUSSION

In *example 1* we see that the dyad’s interaction did not lead to a joint region or attractor that was in the same region as the attractors of both individuals. The couple’s states continuously oscillate between two divergent attractor regions. Each attempt to approximate the participants’ respective attractor zone implied a deviation from the developed zone of the other participant. Each experienced deviation was followed by a strong inclination to avoid the jointly enacted quality and to increase it toward the opposite direction and back to an initial preferred range.

Comparing the couple’s states to the individual attractor region we observe that a greater value of *D* for him is in tension with his preferred zone of well-being that entails lower values of *D*. Yet when the couple’s states show a greater value of *P*, then this implies a tension for her. For both individuals the quality of interaction therefore turns out to be in continuous tension with their individual preference for self maintenance (the preferred balance between *D/P*). The tendencies of the individuals to respond to the tension by fully going back to their own preferred zone of well-being leads to a breakdown of the relationship.

Based on this simple model we hypothesize that individuals whose initial ranges of preferences of distinction and participation are highly opposed are less likely to engage in sustained interactions when for both participants the quality of interaction is in non-negotiable tension to their developed preferences.

In *example 2* a joint region (the saddle) was created based on a partial match of the two individual attractor regions and shorter transients back to original individual attractors. As in the previous example the experienced quality of the interaction (more or less *D* or *P*) perturbates the individual participants’ preferred range and is in tension with their attractors. However, in contrast to *example 1*, the individuals do not fully go back to their initial range *D/P*. Instead they remain within the vicinity of the other’s range of preference. In *example 1* the individuals are affected by the interaction and act in accordance with their own individual goals. In *example 2* the individuals are affected by the interaction (experienced perturbation to *D/P*), but they also adapt their own preference in dependence on and *through* the interaction. As a result, their interaction not only perturbates the individuals’ preferred range but actually alters it. In this example, a higher value of *P* implied staying within some region of higher *P* despite a tension with individual preference for low *P* or, when the interaction showed a higher level of *D* and this was in tension with a developed low value of *D*, it implied approaching a higher *D* than usual. In this way both partners increased the tolerance toward the interaction to act as a gradient on one’s own individually preferred range of *D/P*. This activity allowed for the development of a new, shared zone of preference.

Based on this simple model we hypothesize that individuals who have attractors that show overlap (here, in the dimension of distinction) are more likely able to negotiate tensions caused by perturbations and to jointly adapt their individually developed attractors so that they allow for sustained interactions.

From the dynamical systems conceptualization of the two relationships we derive two styles of negotiation of *D/P*. The first style, corresponding to couple 1, shows that both individuals avoid deviations from their original range of preference. We call this style *passive-closed* as the individuals enter the relationship and react to the tension it creates but do not actively shape the interaction nor adapt their own attractor.

The second style, corresponding to the couple from *example 2*, shows tolerance for perturbation and potential change for both individuals. We call this style *active*-*open* as the individuals enter the relationship and gradually adapt their movement. An experienced tension is not reacted to independently from the ongoing interaction. The active-open style appears to delegate some of the tension caused by perturbation into the individuals’ joint negotiation of *D/P*, creating new, shared spaces of balancing their individually developed ranges of *D/P*.

One can speculate that in this way some interactions can create *corrective experiences* (Castonguay and Hill, 2012) to form new, previously unavailable, evaluation and negotiation strategies, thus effectively changing the individuals’ previously developed attractors.

We hypothesize that the active-open style is more likely to ensure well-being in a relationship, i.e., not only that a relationship is sustained but also that the quality of the interaction can meet the needs of both individuals. That said, we do not suggest that the accommodation of participants’ well-being must always imply an ongoing or actual engagement. There are cases, like *example 1*, where a couple is unable to negotiate the individually experienced tension in a way that still allows for sustaining their relationship. But it is easy to conceive of couples who manage to stay together and are unhappy nevertheless, simply because the negotiation of tension occurs at the expense of the needs of one or both. Negotiation of well-being in a relationship is not only finding continuity in interaction, it is also finding it under specific conditions, namely by considering whether the interaction quality is in tension or in accordance with the individuals’ needs, and their developed preferences for *D* and *P*. Compatibility or well-being therefore does not necessarily require individuals to share high values of participation and remain constantly open and ready to be affected by one another. As *example 2* shows, it may also involve a greater amount of separateness or even periods of disconnection. In the example both partners might need to strongly feel valuable as a person also independently from the other partner or simply wish to spend more time by themselves. This could make them compatible despite a difference in participation preference. This couple can sustain a relationship with actual interactions and some extent of connectivity but also with spaces of disengagement or disconnection, allowing individuals to experience themselves independently from each other^2^.

We should emphasize again that our suggestions apply for close relationships and not for every social interaction. There is an abundance of potential and actual social interactions that are not even remotely considered to be relevant for a person’s self maintenance. Certain types of relationships however, such as romantic relationships, friendships or family bonds, but also some relations dictated merely by cultural agreement such as between employer and employee, are usually considered as fundamentally important or closer than others. Based on our model we can speculate that this is the case precisely because they are considered as important sources for self maintenance and spaces for engaging in the existentially needed *joint* negotiation of both norms of distinction and participation. The more a relationship is deemed to provide such a space the more relevant it will appear. In this sense, being in a relationship is also always an individual choice.

Our account suggests that struggle in a dyadic relationship is in principle unavoidable. This is because any sustained interaction implies that there are two individuals that each have their own goals of social survival and that thus have developed perspectives on how interactions can contribute to them. This leads to constant perturbations that individuals can experience as tension and that can manifest as struggle. Whether or not the couple can maintain the relationship will depend on the individuals’ range of preference and their capacities to tolerate deviations from that range, but also on how the individuals adaptively evaluate and re-evaluate the interaction.

## CONCLUSION

In this paper we conjoined the enactive approach to self with dynamical systems theory to shed light on some basic dynamics underlying struggle and communion in dyadic relationships. We proposed a model of relationship dynamics in terms of a dyadic phase space emerging through the summation of individuals’ phase spaces and assessed struggle or well-being in terms of movements of dyadic states in tension or in harmony with individual attractors. The model predicts that a relationship is sustained when the couple develops a new joint attractor toward which dyadic states tend to move. This is most likely when there is (1) overlap in preferred ranges of distinction and participation in combination with a high estimation of the relationship’s potential to accommodate balancing individual ranges of distinction and participation, as well as (2) an active-open style, in which participants adapt their individual ranges according to their interaction. Because such a relationship has greater potential to meet the needs of both participants to feel more or less connected, and more or less recognized in their own right, it is more likely to lead to well-being.

Presently, we must note some divergences and limitations in conceptualizing an enactive approach to self in terms of DST. In the enactive view, the self is generally co-determined in interaction, and thus already entails perturbations through social interactions. The dimensions of distinction and participation not only mirror the individual’s trajectories but also entail that these trajectories depend on interactions with others. At later stages of the individual’s development, not every interaction matters for self-organization. And yet, at the same time, a self also has developed particular tendencies (dispositions) that constrain to which extent these trajectories are open to perturbations by others, allowing a more flexible evaluation of interactions. Future elaborations on our model have to account for the fact that social interactions and relations matter at different but inextricably linked levels, such as development, dispositional as well as situational enactment of the self. They require clarifications of enactive or dialectical conceptions of identity and the development of corresponding mathematical concepts to arrive at closer approximations for the model and what the model represents. Levins’ work on the relation of dialectical and systemic theory (Levins, 1998) and Van Geert’s DST approach to cognitive development in children (Van Geert, 1998) could serve as inspirations to this end.

Our considerations are exploratory, but we believe they can serve as a starting point to deepen our understanding of the complex interrelation between individual and dyad. They might further help to shed light on interrelations of important phenomena and aspects associated with struggle in dyadic relationships, such as vulnerability and shame, mutual recognition, intimacy, co-dependency, and trauma.

Apart from its potential to assist theoretical integration our proposal may be supported by further quantitative research, for example, through repeated measurements of *D* and *P* preferences of people in a relationship. Methods are available for the assessment of communion and agency (the FAMOS: Grosse Holtforth and Grawe, 2000; the IIP: Horowitz et al., 1994), which may be used as an approximation of the enactive concepts proposed here. It also promises applicability to various empirical fields, for example psychotherapy, and a variety of existing methods of measurement could be used or re-evaluated in light of it.

In this vein, a goal of therapy could be to raise individuals’ awareness that they have existential goals (distinction and participation) that are continuously at play and that affect their interactions. At the same time, they should be encouraged to recognize that this equally applies to the partner and that their relationship is thus a jointly negotiated dynamic of their own individual goals (Stern, 2004). In therapy a couple’s current relationship status could be assessed in terms of individuals’ current *D/P* attractors, how the interactions tend to perturbate them, and the strategies that the couple uses to negotiate these perturbations. This could be complemented by an assessment of individuals’ attractors developed before the relationship *D/P* (e.g., through questionnaires), and by determining the general likelihood of overlap between their attractor regions. For this purpose it might be crucial to evaluate the actual capacities of the individuals for tolerating perturbations and allowing for adaptive change in their developed attractors *D/P*, taking for instance into account factors such as stress level, emotion regulation capacities, attachment styles, past traumata and how they might constitute hindrances (repellors).

Since psychotherapy is itself a dynamic social interaction, it provides a setting in which participants can develop, through active engagement of client/couple and therapist, novel strategies to co-calibrate their self-organization, i.e., narrow the window of oscillation between the opposing attractors or secure a shared zone of well-being. To this end, especially, systemic or interactional approaches to psychotherapy such as the “open-dialogue” approach (e.g., Seikkula and Olson, 2003; Seikkula, 2008) could serve as useful resources. We propose that evaluations and improvements can and should also account for the fact that enactment of relational processes is bodily mediated. Inspiration for reconsidering interventions and assessments in terms of co-negotiation of self maintenance might therefore also come from areas such as mindfulness training, body psychotherapy and dance therapy (e.g., Kabat-Zinn, 1994; Koch et al., 2007; Röhricht, 2009; Tschacher et al., 2014).

Last but not least, from an ethical point of view our proposal is also meant to encourage a greater tolerance for negativity and struggle as necessary aspects of social life. The self individually is a locus of tension and conflicting tendencies: one needs others, and yet at the same time one also needs to feel capable and recognized independently of them. When two people come together the potential for conflict is increased even more. The recognition that we contribute to one another’s self maintenance, and that this is not an easy endeavor, could be a way of affirming the socially existential basis of life as such. Like life, the self resists rigidity. Like life, it is ever moving and not fully determined as long as it exists. Because of this openness of self, relationship struggle must be a necessary aspect of life.

## Conflict of Interest Statement

The authors declare that the research was conducted in the absence of any commercial or financial relationships that could be construed as a potential conflict of interest.

## Footnotes

1 This is not to say that the body does not matter but that individuation does not emerge through bodily processes in isolation. The body changes its status becoming a mediator of the self as socially enacted existence. The bodily sense of self can be biologically grounded but its meaning for the self *as individual being* can only be derived in dependence on how the self is in the world that is, based on and in relation to others. It must be seen as a *matrix* telling us (e.g., through emotions) how we fare in these interactions with regards to the minimal purpose of human existence: to be some*one* who is at the same time a person in her own right while also being a person that one can connect to. Bodily experiences thus acquire a social meaning.

2 As stated in the famous expression “opposites attract,” individuals can also have no overlap at all and still experience each other as compatible because their different attractors may complement one another. And vice versa, individuals might have a great overlap, sharing high participation and low distinction and still struggle because of a lack of feeling acknowledged as being more than a partner for another person.
